# Dynamic Cervical Implants in Patients With Disc Degenerative Disease: A Single-Center Cohort From the Greek Population

**DOI:** 10.7759/cureus.27243

**Published:** 2022-07-25

**Authors:** Triantafyllos Triantafyllou, Alexandros G Brotis, Efthimios Dardiotis, George Fotakopoulos, Kostas N Fountas, Kostas Paterakis

**Affiliations:** 1 Neurosurgery, General Hospital of Lamia, Lamia, GRC; 2 Neurosurgery, General University Hospital of Larissa, Larissa, GRC; 3 Neurology, General University Hospital of Larissa, Larissa, GRC

**Keywords:** cervical, fusion, outcome assessment, dynamic cervical implant, cervical degenerative disorders

## Abstract

Aim

The aim of this study was to review the safety and feasibility, clinical and radiological outcomes, and postoperative complications associated with the use of dynamic cervical implants (DCI).

Patients and methods

A prospective single-cohort study was performed of all consecutive patients who underwent DCI implantation as an adjunct to anterior cervical discectomy. We measured the anterior disc space height (ADH) and posterior disc space height (PDH), as well as the ADH/PDH ratio.

Results

In 11 patients, the ADH/PDH ratio averaged 0.98 (range: 0.7-1.125) postoperatively, from the initial 0.96 (range: 0.72-1.106).

Conclusion

DCI seems to be a viable alternative to anterior cervical discectomy and fusion. However, its role in motion preservation and protection against the degeneration of the adjacent segment is questioned.

## Introduction

Adjacent segment degeneration (ASD) occurs after anterior cervical discectomy and fusion (ACDF), with a reported incidence of 2.9% per year, reaching 25% at 10 years [1]. Both the natural history of the disc degeneration and fusion are considered as causative factors of this phenomenon [1]. ASD is potentially prevented with the use of motion-preserving implants [2].

Nowadays, artificial discs are available for cervical total disc replacement (CTDR), with satisfactory results [3]. The incidence of ASD with CTDR in the treatment of cervical disc disease is less than the equivalent with ACD [2]. However, CTDR has been associated with some significant complications, including heterotopic ossification (HO), implant migration, persistent pain, and device failure [3]. The dynamic cervical implant (DCI) has been designed as a motion-preserving prosthesis in preventing ASD, alternative to CTDR [2]. It is a single-piece, C-shaped implant, with teeth for anterior anchorage, which is inserted via the classical anterior cervical approach [2]. It is promising in maintaining cervical flexion-extension, while preserving the disc height against axial loading [2]. The main indications for DCI included cervical disc herniation with axial neck pain and/or radiculopathy at one to three levels at the subaxial spine [2]. DCI is contraindicated in cases of mechanical instability, osteoporosis, vertebral fractures, and tumors, as well as any pathology that leads to lack of motion in the preoperative dynamic radiographs [2].

There are a few published reports on DCI in patients with cervical disc herniation, myelopathy, and/or radiculopathy [2,4-20]. This study aims to report our experience on patients with DCI for degenerative cervical spondylosis in terms of feasibility and safety (Q1), clinical (Q2) and radiological (Q3) effectiveness, and its associated complications (Q4).

## Materials and methods

Study design

We performed a prospective single-cohort study to evaluate the clinical and radiological outcomes in our population after DCI in patients with cervical degenerative disease. The study took place in our Tertiary Teaching Hospital. No participants’ informed consent was required for our study, as our study was based on anonymized hospital records. All the participants’ data were handled according to the Helsinki Declaration and the Health Insurance Portability and Accountability Act (HIPAA). The manuscript was written based on the PROCESS checklist [21].

Patient selection

Anterior cervical discectomy and DCI implantation were indicated in selected patients with cervical degenerative disc herniation. DCI implantation was contraindicated in patients with osteoporosis, trauma, tumors, and mechanical instability, and in those with documented preoperative lack of motion at the indicated segment. Patients who needed surgery at three or more levels were also excluded. The preoperative examination included cervical plain and dynamic radiographs, and magnetic resonance imaging. All patients were informed about the procedure risks and signed an informed consent.

Surgical technique

The surgical procedure was a standard anterior cervical discectomy [22]. Complete discectomy was performed under fluoroscopic guidance, light microscope, and neurophysiologic monitoring (including motor evoked potential and spontaneous electromyography). The posterior longitudinal ligament was resected in every case, and free disc material was removed whenever it was extruded. The appropriate implant size was defined with the use of trial implants. DCI was inserted slightly compressed, with the depth-stop adjusted to the depth measured from the trial implant. The correct implant position was verified fluoroscopically. The most anterior tooth was located 2- 3mm behind the anterior border of the superior and inferior vertebral bodies. In general, the maximal implant width and height were used for segment restoration. Osteophytes were not removed unless absolutely necessary in an effort to decrease the incidence of HO. Wound closure was performed in the traditional way, and a drain was left in the wound for a single day only if more than one level was treated simultaneously. The patients were mobilized on the first postoperative day using a soft collar to allow for unrestricted but careful neck motion. Anti-inflammatory medications were administered for a seven-day period to reduce the potential for HO.

Feasibility and safety (Q1)

We measured the duration of surgery, the intraoperative blood loss, and the perioperative complication rate to assess the feasibility and safety of the technique.

Clinical outcome measures (Q2)

We used the visual analogue scale (VAS) [23], the Nurick’s scale [24], and the Neck Disability Index (NDI) [25] to assess pain intensity, the associated neurological status, and the neck disability, respectively. Assessments took place preoperatively, immediately after surgery (during hospitalization), and at 3, 6, 12, and 18 months’ follow-up postoperatively.

Radiological outcome measurements (Q3) and long-term complications (Q4)

We measured the anterior disc space height (ADH) and posterior disc space height (PDH), as well as the ADH/PDH ratio from reconstructed computerized (CT) images 18 months after the index surgery. We used dynamic radiographs to visualize the mobility and the fusion status of the index level. Mobility of the segment was recorded when the implant’s opening changed in the dynamic control images. The fusion status was estimated by the presence of at least three bridging trabeculae within the disc space around the implant [26].

Statistical analysis

We used descriptive analysis to describe our population sample. Pre- and postoperative measurements were compared using repeated measures analysis. All statistical analyses were conducted using R (R Foundation for Statistical Computing, Vienna, Austria). Statistical significance was set at 0.05.

## Results

Study sample

**Figure 1:**
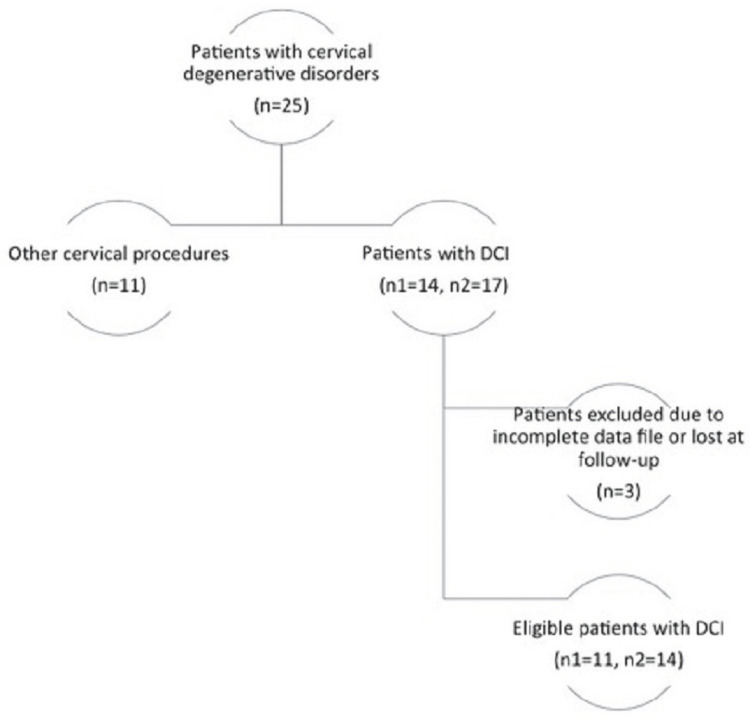
Flowchart of our study sample From the initial 25 patients with cervical spondylosis who fulfilled our eligibility criteria, we implanted DCI in 14 patients as an adjunct to anterior cervical discectomy. Of these, 11 patients with 14 DCIs had complete data files up to the 18 months of follow-up and formed the study sample of our study. DCI, dynamic cervical implant

Eleven patients with a mean age of 47 years (range: 27-56 years) underwent anterior cervical discectomy and DCI implantation at our institution (Figure 1).

Eight patients were male, and three were female. Nine patients presented radiculopathy of the upper extremities, while the remaining two suffered from mild myelopathy (Nurick's grade I). Altogether, 14 DCI implants were used; three patients were operated on at two levels with an equal number of DCI implants. Seven DCIs were implanted in the C6-C7 level, five in the C5-C6, and one in the C3-C4 and C4-C5 levels (Table 1).

**Table 1:** General characteristics of our study population

Characteristics		
Gender	Male	8
	Female	3
Average age in years (range)	47 (range: 27-56)	
Clinical presentation	Myelopathy	2
	Radiculopathy	9
Number of levels	1	8
	2	3
Level involved	C3-C4	1
	C4-C5	1
	C5-C6	5
	C6-C7	7

Feasibility and perioperative safety (Q1)

The duration of surgery averaged 55 minutes (±12 minutes) per level. The estimated blood loss did not exceed 100 cm3 in any case (57cm3 ± 23cm3). No intraoperative complications were recorded.

Clinical outcomes (Q2)

**Figure 2:**
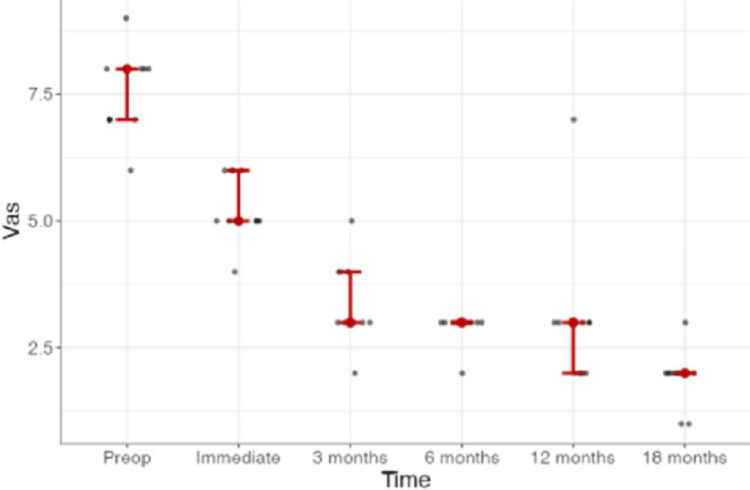
Line graph of radicular pain according to VAS during our study There was marked improvement in arm pain 18 months after the index surgery. VAS, visual analogue scale

The preoperative radicular pain intensity (7.55 ± 0.820), as measured by the VAS, improved at all postoperative measurements (p < 0.001) (Figure 2).

**Figure 3:**
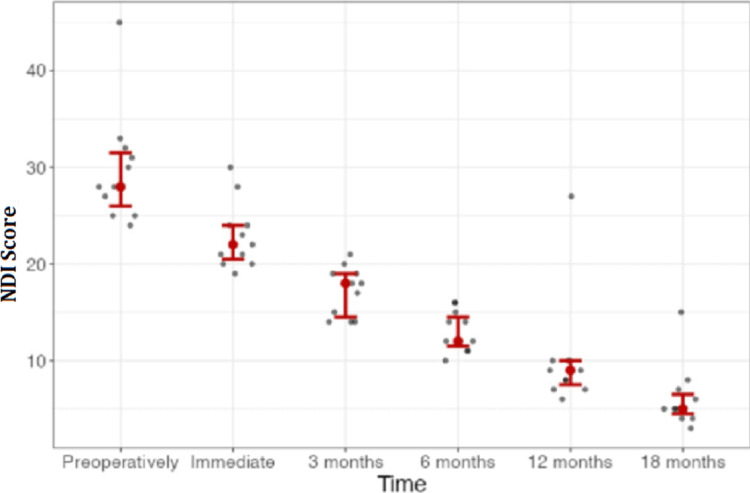
Line graph of neck disability according to NDI during our study There was marked improvement in disability during the 18 months of follow-up. NDI, Neck Disability Index

NDI scores decreased immediately after surgery (Figure 3).

This trend continued at the 3-, 6-, 12-, and 18-month re-evaluation. On the other hand, the mild myelopathy signs, observed in two patients, persisted in the 18 months of follow-up.

Radiographic outcomes (Q3)

**Figure 4:**
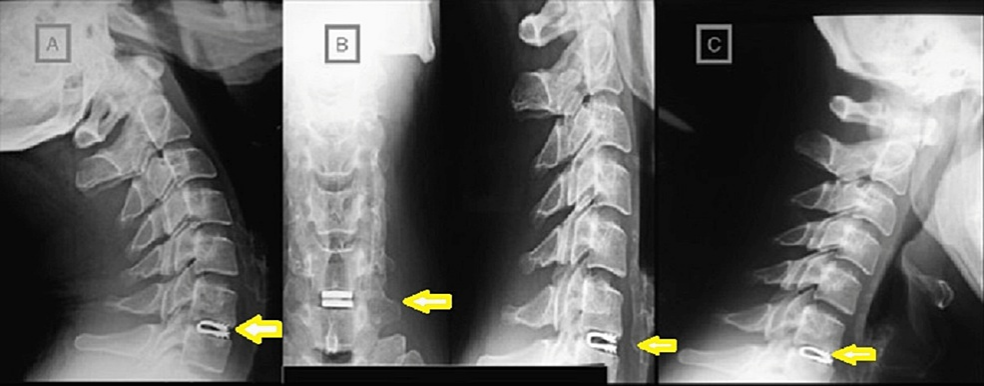
Dynamic (A, C) and plain (B) radiographs of a patient with DCI (arrows) DCI, dynamic cervical implant

**Figure 5:**
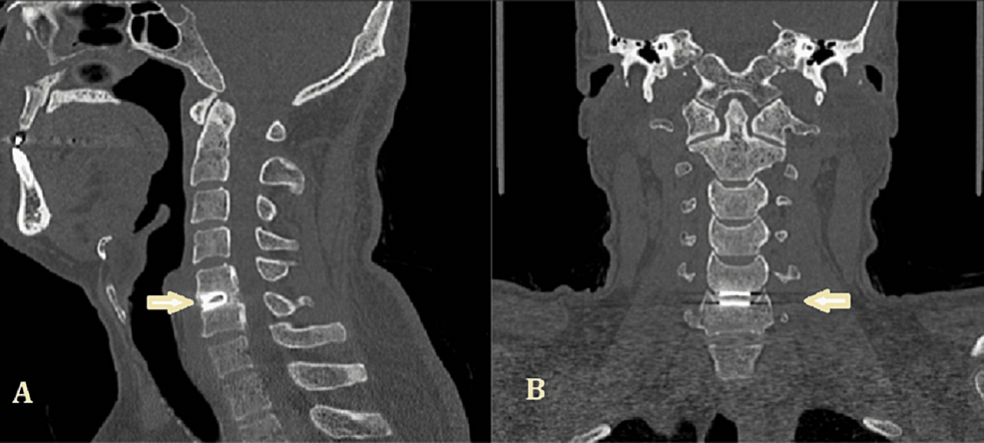
Sagittal (A) and axial (B) CT images of a patient with DCI (arrows) DCI, dynamic cervical implant

All 14 implants were placed in the proper position, as this was verified on the intraoperative and the immediate postoperative radiographic control (plain radiograph). With one exception, the implant’s most anterior teeth were at least 2 mm behind the anterior vertebral rim. From the dynamic radiographs of the third postoperative month, it was noted that the segments’ mobility was maintained. No implant was rotated until the last radiographic follow-up (Figures 4, 5).

The cervical lordosis was restored in all cases postoperatively. Aggregate radiographic results are shown in Table 2.

**Table 2:** Radiological outcome 18 months after anterior cervical discectomy and DCI implantation ADH, anterior disc height; PDH, posterior disc height; DCI, dynamic cervical implants

Parameters	Preoperative	Postoperative
ADH	6.7 mm (range: 5.6-7.1 mm)	7.2 mm (range: 5.8-9.1 mm)
PDH	6.8 mm (range: 6.2-7.3 mm)	7.3 mm (range: 6.6-8.0 mm)
ADH/PDH	0.96 (0.72-1.106)	0.98 (range: 0.7-1.125)
Fused segments	0	8

**Figure 6:**
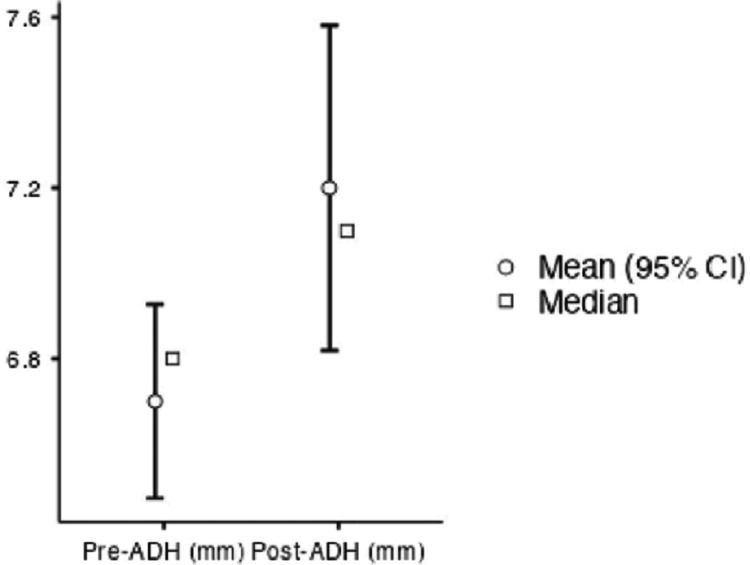
ADH preoperatively and 18 months after the index surgery ADH, anterior disc space height

The average ADH increased from 6.7 mm (range: 5.6- 7.1 mm) preoperatively to 7.2 mm (range: 5.8-9.1 mm) postoperatively (Figure 6).

**Figure 7:**
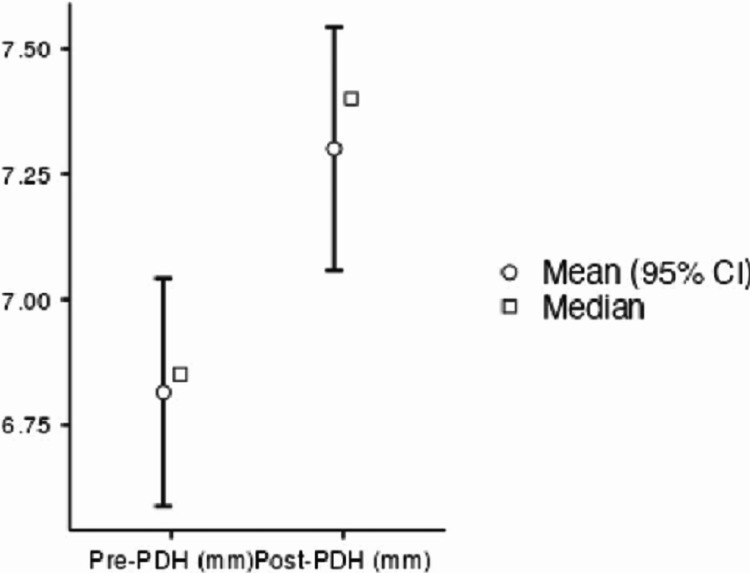
PDH preoperatively and 18 months after the index surgery PDH, posterior disc space height

Similarly, the PDH increased from 6. 8 mm (range: 6.2-7.3 mm) preoperatively to 7.3 mm (range: 6.6-8.0 mm) postoperatively (Figure 7).

**Figure 8:**
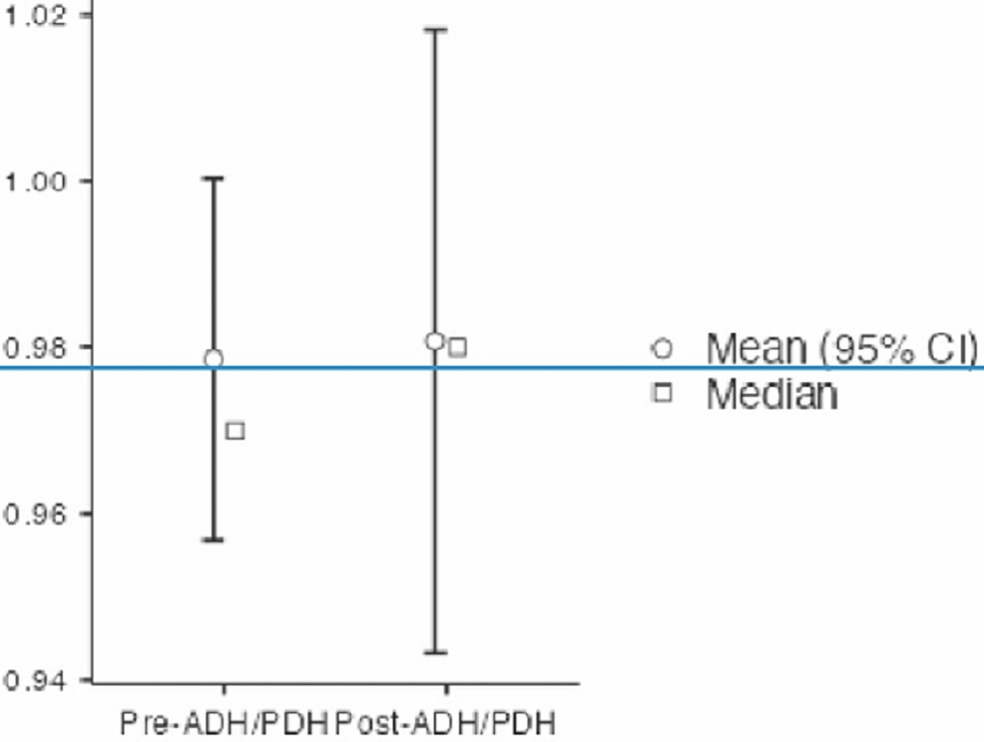
ADH/PDH ratio preoperatively and 18 months after the index surgery ADH, anterior disc space height; PDH, posterior disc space height

Finally, the ADH/PDH ratio averaged 0.98 (range: 0.7-1.125) postoperatively, from the initial 0.96 (range: 0.72-1.106) (Figure 8).

Long-term complications (Q4)

Signs of fusion in some of the index segments were noted in the sixth postoperative month in plain and dynamic radiographic control. Eight segments resulted in multiplanar reconstructed computed tomography (MPRCT)-documented fusion at the end of the 18-month follow-up (Table 2). Nevertheless, fusion did not appear to affect the patients’ clinical outcome. A 35-year-old patient with DCI implants at C5-C6 and C6-C7 levels presented with reemergence of his axial pain at the 12-month follow-up. Plain radiographs showed significant straightening of the cervical lordosis, whereas the MPRCT depicted an impingement of the implants into the inferior endplates, surrounded by a marked translucency. The patient’s symptoms did not respond to conservative management. Finally, in an effort to eliminate segmental motion and achieve solid fusion, both implants were removed and replaced by PEEK cages. The patient’s complaints improved at the 18-month follow-up.

## Discussion

Overview of our findings

There are only a few studies reporting on DCIs, since its initial description as a motion-preserving cervical implant by Matgé in 2002 and its introduction in clinical use by him in 2004 [2]. Almost a decade after DCI initial description, Matgé stated that shock absorption together with maintained motion in the DCI may protect adjacent levels from degeneration in long-term follow-up [10]. In the current study, we realized that DCI is a user-friendly adjunct to anterior cervical discectomy. It increased both anterior and posterior disc heights, opening thus the intervertebral foramina, while it maintained the proper cervical lordosis. As a consequence, the patient’s pain intensity decreased with a marginal improvement in the neck-related disability in the long run. However, the implants did not seem to preserve the segmental mobility in the anteroposterior axis. The perioperative morbidity associated with the use of DCI was minimal.

Biomechanical background

Evidence from laboratory studies supports that the use of DCI is characterized by near-normal biomechanical properties. In particular, Welke et al. tested the biomechanical properties of DCI in sheep models [15,16]. The authors concluded that the implant showed a tendency to stabilize the segment, while allowing some degree of residual mobility in the flexion and extension, whereas it significantly reduced movement and thus stabilized the affected segment in lateral bending [15,16]. Similarly, in the adjacent segments, the kinematics were not significantly affected in the three directions tested [15,16]. The only significant change observed was an increase of intradiscal pressure of the cranial disc in flexion [15,16]. In another study, Mo et al. compared the biomechanical effects of the standalone U-shaped configuration on the cervical spine with a sliding articulation design (Prodisc-C) and an anterior fusion system using a finite element study [8]. The study concluded that the U-shaped implant was superior to the other implants in maintaining the spinal kinematics and imposing minimal influence on the adjacent soft tissues [8].

Safety and effectiveness

A number of studies have reported similar results to our findings regarding the safety and effectiveness of the DCI as an adjunct after anterior cervical discectomy [2,6,10] (Table 3).

**Table 3:** Review of the literature relevant to DCI R, retrospective; CS, case series; NR, not reported; P, prospective; Coh, cohort; ACDF, anterior cervical discectomy and fusion; CTDR, cervical total disc replacement; DCI, dynamic cervical implants

Author	Year	Design	Enrollment period	Country	N	Age	Levels	Pathology	Follow-up (months)	Comparison
Matgé [10]	2012	R / CS	NR	Belgium	44	NR	﻿1-, 2-, or 3-level	Cervical degenerative disc disease	6 to 24 months	No
Eldin and Mohamed [2]	2014	P / CS	2009-2012	Egypt	15	47	Single	Cervical degenerative disc disease	12	No
Li et al. [6]	2014	P / Coh	2009-2011	China	39	45	Single	Cervical degenerative disc disease	26.7	ACDF
Wang et al. [14]	2014	R / CS	2010-2010	China	30	56	Single	Cervical degenerative disc disease	26	No
Zhu et al. [18]	2014	R / Coh	2009-2011	China	25	47	Single	Cervical spondylotic myelopathy	20	ACDF, CTDR
Matgé et al. [9]	2015	P / Multicenter	2008-2011	Germany and Luxemburg	175	47	﻿1-, 2-, or 3-level	Cervical degenerative disc disease	26	No
Matgé et al. [11]	2015	P / CS	2008-2011	Luxemburg	53	50	﻿1-, 2-, or 3-level	Cervical degenerative disc disease	24	No
Richter et al. [12]	2016	P / Coh	2009-2010	﻿Switzerland	30	44	﻿1- or 2-level		12	ACDF
Shichang et al. [13]	2016	R / Coh	2010 - 2012	China	67	42	Single	Cervical spondylotic myelopathy	24	CTDR
Li et al. [7]	2018	R / Coh	2009-2013	China	35		Single	Cervical degenerative disc disease	74	ADCF
Zhu et al. [20]	2018	R / Coh	2009-2011	China	43	47	Single	Cervical degenerative disc disease	71	ACDF
Eldin and Mohamed [4]	2018	P / Coh	2012-2015	Egypt	15	47	Single	Cervical degenerative disc disease	22	ACDF
Wang et al. [17]	2018	R / CS	2010	China	38/42		1- or 2-level	Cervical degenerative disc disease	73	No

Eldin and Mohamed performed a prospective study to evaluate the surgical safety and effectiveness of DCI in 15 patients with single-level cervical disc disease [2]. The use of DCI was associated with minimal blood loss and short hospital stay, without any major perioperative complications [2]. There was a significant improvement in the neck and myelopathy symptoms in 87% and 50% of the cases, respectively [2]. On the other hand, Li et al. compared DCI to ACDF in a two-year cohort study by using clinical and radiological parameters, focusing on the index and adjacent segments [6].

The authors found no definitive evidence that DCI implantation was associated with better intermediate-term results than ACDF [6]. The incidence of adjacent segment changes was reported to be as high as 12.8% two years after the DCI placement [6].

Radiological outcomes

It has been documented that DCI maintains the height of the index level successfully [10]. In addition, the mobility of the index level appeared to be preserved as visible motion on postoperative flexion and extension dynamic views [2]. Wang et al. reported good clinical and radiographic outcomes after studying 30 patients with single-level degenerative cervical disc disease and treated with DCI for an average of two years [14]. According to their experience, the intervertebral disc height was slightly increased after surgery [14]. On the contrary, the range of motion (ROM) initially decreased but reached the preoperative values two years after surgery [14].

Finally, the authors noticed a linear correlation between motion of the adjacent vertebral endplate and motion within the implant [14]. Another multicenter study with 175 patients postulated that the ROM at the level treated with DCI was slightly reduced, but no significant changes could be verified at the adjacent levels [11]. In our current study, we focused on the ADH, PDH, and the ADH/PDH ratio; thereby, DCI seems to restore the disc height of the index level, while correcting the cervical lordosis.

Complications

Matgé et al. studied the clinical and radiological outcomes of DCI implantation in 53 patients for one-, two-, or three-level degenerative cervical disc disease with a mean follow-up of 24 months [9]. The authors noticed HOs in 21 (60%) patients, asymptomatic implant migration in one (3%) patient, implant subsidence in two (6%) patients, and symptomatic adjacent disc degeneration in one (3%) patient [9]. In a similar study by Wang et al., the implant migrated forward in 10 (23.8%) of 42 cases, whereas HOs were detected in 24 (57.1%) of the 42 DCI segments [17]. Subsidence was observed in 14 (33.3%) of 42 DCI segments, and two patients experienced symptom recurrence, for which no further surgical treatment was required [17]. In our cohort, symptomatic implant subsidence was noticed in one patient with two DCI implants and required reoperation with fusion of the index segments. In a comparison with ACDF and CTDR, DCI seems to be as safe as effective as ACDF and CTDR. Zeng et al. performed a meta-analysis to compare the safety and efficacy of DCI versus ACDF and CTDR in treating cervical degenerative disc disease [19]. Based on a total of six studies with 491 patients, the authors observed no statistical difference in regard to the operative time, intraoperative blood loss, Japanese Orthopedic Association score (JOA), VAS, and NDI between the DCI and ACDF groups, or the DCI and CTDR groups [19]. However, compared with the ACDF group, the DCI group presented with higher treated segmental ROM, lower cephalic segmental ROM, and caudal segmental ROM, but equal in overall ROM. No significant difference in cephalic, treated, and caudal segmental ROM was recorded between the DCI group and the CTDR group [19]. In addition, there were no differences in regard to the complication rates between DCI, ACDF, or CTDR [19]. Zhu et al. compared a group of 43 patients with DCI and an equal number of ADCF. ASD was observed in seven (16.3%) patients in the DCI group and nine (20.9%) patients in the ACDF group (p = 0.579) [20]. Similarly, Shichang et al. compared a group of 67 patients with DCI and 85 patients with CTDR [13]. Overall, 15 (22.3%) patients in the DCI group and 24 (28.2%) patients in the CTDR group developed HO 24 months after surgery (p = 0.739) [13]. ASD was observed in nine (13.4%) patients in the DCI group and in 13 (15.3%) patients in the CCTDR group (p = 0.734) [13].

Limitation

There are several limitations in the current study. Our study sample is relatively small to reach generalizable results. In addition, the population in the study is highly selected. We studied the implant’s radiological and clinical outcomes in young patients with minimal myelopathy. Patients with traumatic or neoplastic lesions were intentionally excluded. The follow-up is limited to 18 months. Future randomized large-scale multi-cohort studies are needed to find the actual role of DCI in our surgical armamentarium for managing degenerative spinal disorders.

## Conclusions

DCI seems to be a viable alternative to ACDF and CTDR. It is easy to use, and there is evidence that it maintains the segmental disc height and the ROM in flexion/extension. The patient’s pain intensity and spinal cord related disability both may improve in the mid-term follow-up. However, its role in motion preservation and in protecting the adjacent segment is questioned and requires further elucidation in larger and statistically powerful studies.
